# Whims of Hysteria

**Published:** 1885-02

**Authors:** 


*Whims of Hysteria.*—A young woman, twenty-five years old, hysterical, during 1882 had an attack of acute articular rheumatism, from which she recovered. In 1883, she complained of sharp pains in the right knee. There was noticed, a little below the joint, a brownish swelling where the head of a pin could be felt. At the same time several other swellings were discovered in different parts of the body and especially on the right and left arm, on the chest, abdomen, legs, etc. During the month of December there were extracted 65 entire needles and 6 broken ones; in January 110, and, finally, the patient confessed to have swallowed five packages containing 140 needles. This case was reported by Professor Wide in the *Révue Medicale*.

A similar case was told by Herboldt (1822) of a girl fourteen years old, who, in 1807 had colics, hysterical attacks, etc. In the year 1819 numerous abscesses appeared on the surface of the body, from which were extracted 273 needles; in 1820 she had paralysis of an arm; in 1821 a hundred needles were extracted, and in 1822, while in the hospital, 32 needles were again taken from her body. Human whims!

